# Virological Insights from ARC-520 siRNA and Entecavir Treated Chronically HBV-Infected Patients and Chimpanzees

**DOI:** 10.3390/microorganisms13081787

**Published:** 2025-07-31

**Authors:** Christine I. Wooddell, Lung Yi Mak, Wai-Kay Seto, Bruce D. Given, Man-Fung Yuen

**Affiliations:** 1 Arrowhead Pharmaceuticals Inc., 502 S. Rosa Road, Madison, WI 53719, USA; cwooddell@arrowheadpharma.com; 2 Department of Medicine, State Key Laboratory of Liver Research, School of Clinical Medicine, The University of Hong Kong, Hong Kong, China; loeymak@gmail.com (L.Y.M.); wkseto@hku.hk (W.-K.S.); 3 Arrowhead Pharmaceuticals Inc., 177 E. Colorado Boulevard, Suite 700, Pasadena, CA 91105, USA; bgiven@arrowheadpharma.com

**Keywords:** hepatitis B virus transcription, hepatitis B virus RNA, cccDNA, HBV X, HBV siRNA, RNA interference therapeutics

## Abstract

In a previous study, eight chronically HBV-infected nucleos (t)ide analog (NA)-naïve patients began receiving entecavir (ETV) concomitant with a single ARC-520 HBV siRNA injection. This single dose of ARC-520 (SD) was followed by 6–8 months of ETV alone before the patients received 4–9 monthly doses of ARC-520, the multi-dose (MD) period, while continuing ETV. Quantities of HBV DNA, RNA, and antigens were measured from serum and a liver biopsy collected ~30 months after the last MD from five patients. All full-length HBV transcripts from the livers were characterized. Viral parameters and HBV transcripts from patients were compared to these measurements collected at multiple points in ARC-520 + ETV-treated chronically HBV-infected chimpanzees. Multiple forms of HBx mRNA were observed, and these differed between chimpanzees and patients. Products of cccDNA were greatly decreased in patients who were previously highly viremic and HBeAg+, although a biopsied patient had similar amounts of cccDNA to the highly viremic HBeAg+ chimpanzees. The comparison of all HBV transcripts and cccDNA levels between patients and chimpanzees demonstrate the transcriptional silencing of cccDNA following the siRNA treatment of patients but not the chimpanzees that received a different treatment regimen. Results from this small study suggest that continued NA treatment during and between periods of HBV antigen re-expression post-siRNA treatment enhanced viral parameter reductions.

## 1. Background

Approximately 256 million people globally are chronically infected with the hepatitis B virus and 1.1 million annually die of resulting liver disease or hepatocellular carcinoma [1,2,3]. Chronic hepatitis B infection (CHB) is transmitted through blood, primarily due to perinatal infection, as more than 90% of people infected after the age of five can immunologically control the virus, which is a “functional cure”. The prompt HBV vaccination of the newborns of HBV-infected mothers has greatly decreased but not eliminated the incidence of infants becoming chronically infected [4]. CHB is determined by the presence of serum HBV S antigen (HBsAg) at two points in time at least six months apart. A functional cure is not a true cure of infection because traces of HBV DNA remain in the hepatocytes after HBsAg loss [5].

HBsAg is produced by the HBV covalently closed circular DNA (cccDNA), the genetic minichromosome. cccDNA encodes the viral genome as pregenomic RNA (pgRNA) and all the viral proteins: HBsAg in three forms (large, middle and small HBsAg), HBV e antigen (HBeAg), HBV core antigen, HBV polymerase, and HBV X protein (HBx). All the HBV transcripts that form these viral products have a common 3-prime terminus with a polyadenylation signal (polyA) that is typically 82 bases downstream of the Direct Repeat 1 replication element (DR1). The viral genome is formed within a capsid of core protein by the reverse transcription of the pgRNA to form a partially double-stranded circular DNA. The DNA-containing capsid is enclosed in an envelope of lipids and HBsAg and secreted as the virion. HBV RNA in the serum is primarily enveloped, core-encapsidated pregenomic RNA (pgRNA), or splice products of pgRNA [6].

HBsAg is also produced by HBV DNA that has integrated (iDNA) into the host chromosomes [7,8,9]. While the HBV cccDNA can integrate at any breakpoint within the circular genome, the greatest source of iDNA is from double-stranded linear DNA (dslDNA) that results from mispriming of the (+) strand synthesis during replication [10]. When it is formed, dslDNA has a DR1 element at both ends of the linear DNA, but during integration, the ends of the DNA are often chewed back by nucleases. The promoters and full open reading frames (ORF) for HBsAg (large, middle, and small) are contained in the iDNA, but not the full coding sequences for the pgRNA, HBeAg, core, or polymerase. Cryptic polyAs in the host genome serve to terminate most HBsAg transcripts of HBeAg-negative (HBeAg−) chronically HBV-infected chimpanzees [7]. The promoter and most, but not all, of the coding sequence for HBx are contained in iDNA. An alternative polyA in the HBV sequence 45 bases upstream of the HBx stop codon is often utilized to produce truncated HBx from iDNA in HCC patients [11]. Some HBsAg transcripts from iDNA also utilize this alternative polyA [7,12].

The amounts of cccDNA are higher in HBeAg-positive (HBeAg+) patients than in HBeAg− patients, whereas the amounts of iDNA are higher in HBeAg− than HBeAg+ patients [7,8,9,13,14,15]. HBsAg expression is maintained from iDNA even as the HBsAg expression from cccDNA declines [12,15].

RNA interference (RNAi) therapeutics are a promising modality to achieve a functional cure in CHB patients [16]. ARC-520 was a first-in-class HBV RNAi therapeutic. A single-dose (SD) phase 1b clinical trial (Heparc-2001) began in 2014 to assess the safety, tolerability, and pharmacological effect of ARC-520 in conjunction with continued daily nucleos (t)ide analog (NA), which was entecavir (ETV) or tenofovir [7,17]. The safety, tolerability, and pharmacological effect of ARC-520 were assessed concomitantly in nine chronically HBV-infected chimpanzees. The deep HBsAg reductions in HBeAg+ chimpanzees compared to the more modest HBsAg declines in HBeAg− chimpanzees prompted us to enroll NA-naïve patients in the Heparc-2001 clinical trial. The previously NA-naïve HBeAg+ patients responded similarly to the HBeAg+ chimpanzees with deep, ~1.5 log_10_ HBsAg, and HBeAg reductions [7]. The molecular analysis of the liver HBV DNA and RNA from the liver biopsies of the chimpanzees led to the discovery that most of the HBsAg in the HBeAg− chimpanzees was produced from iDNA. ARC-520 was not able to reduce all the HBsAg transcripts from iDNA because the two ARC-520 siRNA target sites near the DR1 element were often lost in transcripts from iDNA. The treatment of HBeAg− chimpanzees with an HBV siRNA named siHBV-75 that targeted within the HBsAg ORF demonstrated that the HBsAg produced from the transcripts of the iDNA could be deeply reduced when the siRNA target site was present in the mRNA.

The 51 patients in the Heparc-2001 trial had either been receiving NA daily for at least one year prior to enrollment or began NA treatment concomitant with the first injection of ARC-520. Eight of the previously NA-naïve patients, three that were HBeAg+ and five that were HBeAg−, enrolled in a multi-dose extension study of Heparc-2001 (NCT 02065336) [18]. Six to eight months elapsed between the SD phase and the start of the multi-dose (MD) extension study, during which time all patients continued to receive ETV. Patients in the MD study were scheduled to receive 12 doses of ARC-520 given once every four weeks (Q4W), but the development of ARC-520 was terminated during this period due to a toxicity finding in a cynomolgus monkey toxicology study. The toxicity was due to the excipient EX1 and not the siRNA. As a result, the patients received four to nine doses of ARC-520. Liver biopsies were collected from five of the patients at a single time point, and the last follow-up (LFU) that was 28.9–31.1 months after the last MD ARC-520 treatment. ETV was discontinued in only two patients, one who was initially HBeAg+ and one who was HBeAg−, one year after their HBsAg loss.

The chimpanzees in our study were located at two separate sites; here, we compile the viral measurements in serum and liver from six of the seven chimpanzees from the same site that had more similar time points. The seventh chimpanzee had very low serum HBV DNA and HBsAg and was, thus, excluded in the current comparison. As previously described, chimpanzees received daily oral ETV for a lead-in period of 8–20 weeks to reduce viral replication prior to the start of the Q4W intravenous injections of ARC-520 [7]. Chimpanzees received six to ten Q4W doses of ARC-520. After receiving ARC-520, the HBeAg− chimpanzees that responded to ARC-520 with less HBsAg reduction were given one to four Q4W doses of siHBV-75, as previously described [7,12]. Two chimpanzees received a final dose of siHBV-75 plus siHBV-74 (the latter being one of the siRNAs in ARC-520) to develop ARC-521 that would target cccDNA-derived and iDNA-derived transcripts. ARC-521 included the same excipient EX1; thus, patient studies with ARC-521 were terminated at the same time as studies with ARC-520. The last dose of ETV was given to the chimpanzees 9–14 days after the final siRNA injection. The study design was to dose all animals 12 times with ARC-520, but this design was changed during the study to investigate the alternative siRNA in the HBeAg− chimpanzees that responded more modestly to ARC-520. All treatment was ended ahead of schedule to monitor the chimpanzees off treatment for as long as allowed in anticipation of the chimpanzee reclassification by the U.S. Department of Fish and Wildlife. Consequently, chimpanzees with the highest serum HBV DNA received fewer doses of ARC-520 than those with lower HBV DNA. All sample collections from the chimpanzees were conducted prior to 14 September 2015, when the U.S. Department of Fish and Wildlife reclassified as endangered chimpanzees born in captivity at research centers.

Full-length transcripts with HBV sequence were characterized from the liver biopsies of six chimpanzees and five of the eight Heparc-2001 extension study patients, providing insights into HBV virology and patient responses [12]. Subsets of data from the patient extension study that monitored patients for approximately four years have been presented in five publications that have extensive supplementary materials [7,12,18,19,20]. Similarly, subsets of data from the chimpanzee study were presented in three publications [7,12,19]. The aim of this paper was to highlight insights from the patients and chimpanzees that received MD ARC-520 and compare the outcomes.

## 2. Details of the Previous Studies

### 2.1. Materials

As previously described, ARC-520 comprised cholesterol-conjugated HBV siRNAs siHBV-74 and siHBV-77 at a 1:1 ratio [21]. The siHBV-74 target site (HBV position 1779–1797 relative to accession number V01460) overlapped the alternative polyA in the HBx coding region and the siHBV-77 target site (position 1825–1843) overlapped the HBx stop codon. The siHBV-75 target site was within the S gene ORF (position 380–398). siRNAs were intravenously infused with an equal mass of the N-acetylgalactosamine (GalNAc)-targeted melittin-like peptide excipient, EX1, as previously described [7].

### 2.2. Chimpanzee Study

The ETV lead-in prior to ARC-520 dosing was 8 weeks for chimpanzees A4A014, 88A010, and 95A010; 12 weeks for chimpanzee A2A004; and 20 weeks for the most viremic chimpanzees, A3A006 and 89A008. Chimpanzees A2A004, A3A006, A4A014, 89A008, 88A010, and 95A010 received 9, 7, 10, 6, 7, and 7 Q4W doses of ARC-520, respectively. Following MD ARC-520, the HBeAg− chimpanzees that responded to ARC-520 with less HBsAg reduction, 88A010 and 95A010, received three Q4W doses of siHBV-75, followed by one dose of siHBV-75 + siHBV-74, as previously described [7,12]. Chimpanzee 89A008 that became HBeAg− during the ETV lead-in and was, thus, identified as “transitional”, received one dose of siHBV-75 following MD ARC-520. The last dose of ETV in all chimpanzees was given 9–14 days after the final siRNA injection.

### 2.3. cccDNA Measurement

A single needle-biopsy of the liver was collected from the patients and periodic needle biopsies of the liver were collected from chimpanzees to measure liver HBV DNA and HBV RNA, as previously described [7,12]. The real-time quantification of intrahepatic HBV cccDNA from patients was performed as previously described [22,23] with the slight modification that Exonuclease I and Exonuclease III were used in place of Plasmid-Safe DNase [12,24]. The real-time quantification of intrahepatic cccDNA from chimpanzees was performed as previously described using the Plasmid-Safe, ATP-dependent DNase kit [12]. HBV cccDNA from chimpanzees was measured as copies/µg total liver DNA, whereas HBV cccDNA in human patients was measured as copies/cell. To compare copies/µg chimpanzee HBV cccDNA to human HBV cccDNA, we used the conversion factor of 6.5 pg total DNA per cell [25].

## 3. Summarized Observations

### 3.1. Transcriptional Silencing of cccDNA

As shown in Table 1, two HBeAg+ patients of the Heparc-2001 extension study (patients 708 and 710) who were initially highly viremic (>8 log_10_ IU/mL serum HBV DNA) and immune-tolerant achieved serum levels of HBV DNA, HBV RNA and HBeAg (all products of cccDNA) that were either undetectable or below the lower level of quantitation (<LLOQ) at the last follow-up (LFU). The most important finding from the analyses of patient samples was that the mechanism whereby the expression of these HBV products was lost was through the transcriptional silencing of the cccDNA. The silencing of cccDNA transcription followed finite siRNA treatment in conjunction with ETV. The female patient 708 whose HBsAg was 34,388 IU/mL at baseline and <LLOQ at LFU did not have a biopsy, but the male patient 710 whose HBsAg was 80,918 IU/mL at baseline and 25.8 IU/mL at the LFU did. The latter patient had 3.4 copies cccDNA/cell, but this patient had very few detected transcripts from cccDNA in the liver (a total of seven transcripts) and none were pgRNA. Prior to the start of the treatment, HBeAg+ and HBeAg− patients had quantifiable serum HBV DNA, which contains products of cccDNA transcription. At LFU, however, the cccDNA-derived HBV core antigen was not detected in any of the biopsied patient liver tissue, suggesting the transcriptional silencing of cccDNA in HBeAg− patients as well [18].

None of the patients that received liver biopsy were HBeAg+ at the LFU and all had fewer than one copy pgRNA/cell determined by real-time PCR [18]. Thus, few products of cccDNA were detected post-treatment in these patients. There were no pre-study liver biopsies in the patients for comparison. The companion chimpanzee study, however, included multiple liver biopsies of the chimpanzees that provide a reference for the types and quantities of HBV transcripts in CHB individuals prior to ARC-520 treatment [12].

The HBeAg+ chimpanzees A2A004 and A3A006 with similar amounts of cccDNA to the HBeAg+ patients, by contrast, had approximately 2000 cccDNA-derived transcripts that comprised 94–97% of their total transcripts with the small remaining percentage being derived from iDNA (Table 2). The transcription of cccDNA was not silenced in these highly viremic chimpanzees. Unlike the patients, the chimpanzees were not treated with months of continuing ETV during the antigen re-expression period after siRNA treatment nor continued ETV post-MD. The chimpanzees stopped receiving ETV only 1–2 weeks after the last MD ARC-520, which was prior to antigen rebound. In contrast, the patients all had two periods of antigen rebound while on continuing ETV: first after the SD ARC-520 and then again after MD ARC-520.

### 3.2. Extended Entecavir Treatment to Consolidate the Benefits of siRNA Treatment

**Figure 1:**
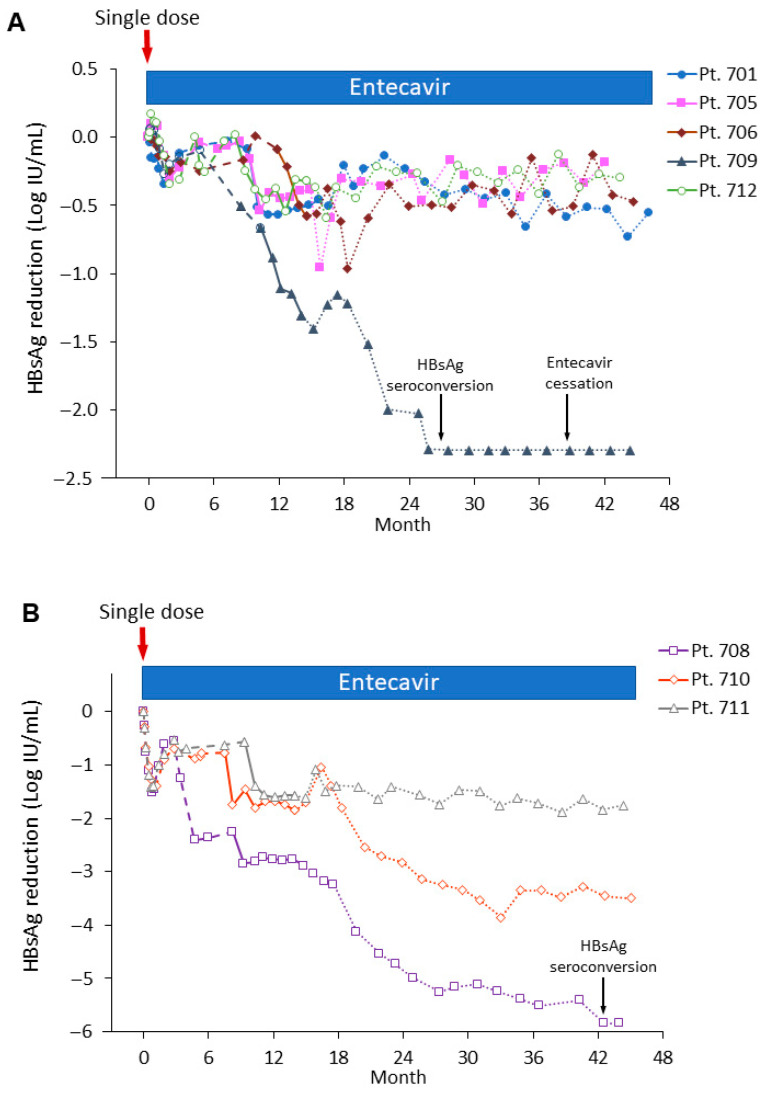
Kinetics of HBsAg reduction after single-dose (SD) and multi-dose (MD) ARC-520 in 5 HBeAg− patients (**A**) and 3 HBeAg+ patients (**B**). Dashed lines represent the follow-up period after the SD prior to start of MD ARC-520; solid lines represent period of receiving MD ARC-520; dotted lines represent a follow-up period after the MD. All patients remained on ETV during the study except, as indicated, for patient 709, who seroconverted for HBsAg. Pt, patient. Reproduced from [18] with permission from BMJ Publishing Group.

An important difference in the treatment of the patients relative to the chimpanzees was that patients continued ETV treatment for at least 30 months after the end of MD siRNA treatment, whereas the chimpanzees discontinued ETV treatment 1–2 weeks after the last siRNA treatment. During these 30 months post-siRNA but on continued ETV, serum viral parameters in patients 708 and 710 continued to decline: HBsAg, HBV RNA, HBeAg, and HBV core-related antigen (HBcrAg), in addition to the expected decline in HBV DNA from the ETV (See Figure 1 and [18]). Although HBsAg could be derived from cccDNA or iDNA, the HBV RNA, HBeAg, and HBcrAg are products of cccDNA. Such declines would be highly unusual for ETV treatment alone in immune-tolerant patients, but they suggest the importance of continued NA treatment to obtain the full benefit post siRNA treatment when HBV antigens become re-expressed. HBsAg continued to decline during this period in HBeAg− patient 709 as well (Figure 1A). Consolidation therapy to inhibit new infection for approximately one year after HBsAg loss is at the discretion of the investigator. HBeAg+ patient 708 and HBeAg− patient 709 discontinued ETV treatment and remained seronegative for HBsAg.

In addition to the benefits of continued ETV treatment post-MD siRNA, patients 708 and 709 demonstrated the beneficial effect of continued ETV between SD and MD ARC-520 (Figure 1A,B). At approximately three months after the SD ARC-520, HBsAg and the other viral antigens rebounded almost to baseline in patient 708, but then began to decline in the five following months without additional siRNA [18,19]. HBsAg decreased further during MD ARC-520 and then continued to decline until the patient seroconverted for HBsAg (Figure 1B). HBsAg rebounded partially in HBeAg+ patient 710 after the SD, appearing to reset HBsAg expression at a lower level than pre-study. Then, following the MD period, his HBsAg again rebounded partially, followed by a decline that continued for ~16 months. A similar pattern of HBsAg decline was observed in HBeAg− patient 709 who lost HBsAg, a functional cure. Although she did not seroconvert, the third HBeAg+ patient 711 had significant decreases in all viral parameters at LFU: −1.76 log_10_ IU/mL HBsAg, −3.13 log_10_ PEI U/mL HBeAg, −2.4 log_10_ kU/mL HBcrAg, −1.4 log_10_ U/mL HBV RNA, and −4.85 log_10_ IU/mL HBV DNA. Altogether, these results suggest the changing viral antigens are detected by the immune system while replication is suppressed.

### 3.3. Change in HBV Transcription During HBeAg-Seroconversion of Chimpanzees

**Figure 2:**
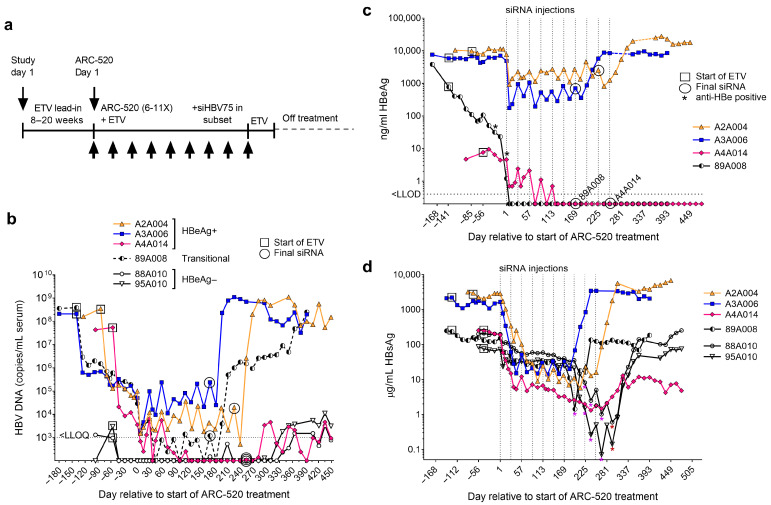
Response to the multi-dosing (MD) of chimpanzees with HBV siRNAs. (**a**) Chimpanzees received daily ETV beginning on study day 1 for an ETV lead-in period of 8–20 weeks, followed by MD with 6–10 monthly intravenous injections of ARC-520 (indicated by arrows below time line) beginning on ARC-520 day 1 (after ETV lead-in) with continued ETV treatment. Some chimpanzees received injections of siRNA siHBV-75 in the S gene open reading frame or siHBV-75 + siHBV-74 after the ARC-520 MD, as described in the Methods. siHBV-74 is one of the X siRNAs in ARC-520. ETV treatment continued until 9–14 days after the final siRNA dose. (**b**–**d**) HBeAg+ chimpanzees (A2A004, A3A006, and A4A014), HBeAg-transitional (chimpanzee 89A008) and HBeAg− chimpanzees (88A010 and 95A010) had a pre-study health check (HC) 40 days prior to study day 1, at which time the ETV lead-in (box) began. Q4W dosing with ARC-520 commenced on ARC-520 Day 1. The timing of the final siRNA injection, either ARC-520, siHBV-75 or siHBV-75 ± siHBV-74 is indicated by a circle. Serum HBV DNA (**b**), HBeAg (**c**) and HBsAg (**d**) measurements are shown relative to Day 1 of ARC-520 treatment. HBsAg (**d**) nadirs following each dose of siHBV-75 or siHBV-75 + siHBV-74 in the chimpanzees that received these siRNAs following ARC-520 MD are indicated by purple or dark red asterisks, respectively. Reproduced from [12] under the terms of the Creative Commons Attribution License.

Two of the initially HBeAg+ chimpanzees seroconverted for HBeAg: a 24-year-old male (89A008) and a 10-year-old female (A4A014) that had 8.6 and 7.7 log_10_ copies/mL serum HBV DNA (equivalent to 7.9 and 7.0 log_10_ IU/mL, respectively) prior to ETV treatment (Figure 2). Chimpanzee 89A008’s HBeAg level decreased by 80% between pre-study HC and the first dose of ETV, thus he was “transitional” for HBeAg [7]. He became HBeAg− during 20 weeks of ETV treatment alone. It has been known that HBeAg− patients have fewer transcripts from cccDNA than from iDNA, and the iDNA-derived transcripts produce HBsAg [7,8,9,15]. Our characterization of the HBV transcripts of chimpanzee 89A008 demonstrated that those from iDNA, initially 33% of all HBV transcripts, were maintained with little change in number during HBeAg seroconversion, but the cccDNA-derived transcripts decreased 81% during the 20 weeks this chimpanzee transitioned to HBeAg-negativity. As previously shown, the siRNAs in ARC-520 were not able to reduce all the HBsAg from iDNA because the siRNA target sites near the DR1 element were often lost in transcripts from iDNA [7]. Thus, this chimpanzee maintained significant HBsAg expression during ARC-520 treatment and did not have the benefit of continued ETV treatment post-siRNA. His serum HBV DNA remained approximately 2 log_10_ copies/mL reduced for about four months off all treatment, but then rebounded to viremia as high as prior to treatment. His end-of-study HBsAg levels seven months off all treatment were only 25% lower than prior to treatment and his ALT was 3 × ULN.

Chimpanzee A4A014 had low but stable HBeAg expression prior to starting ETV treatment and seroconverted for HBeAg after five doses of ARC-520 (Figure 2c). Among her HBV transcripts, 91% were cccDNA-derived. Although she did not have the benefit of continued ETV treatment post-siRNA, she was able to maintain serum HBV DNA <LLOQ, 0.68 log_10_ µg/mL HBsAg, and normal ALT at seven months off all treatment, the last point at which serum was collected [7,19]. Her HBV transcripts were 99% reduced at the final liver biopsy that was 98 days after the final dose of siRNA (ARC-520).

Both chimpanzee A4A014 and chimpanzee 89A008 had 0.03 copies cccDNA/cell as replication began to rebound off all treatment, but they had very different viremia outcomes by study day 533. Chimpanzee A4A014 that had primarily cccDNA transcripts had transitioned to the immune control or low replicative phase of HBeAg− chronic infection (previously known as inactive carrier state), whereas chimpanzee 89A008 expressing more HBsAg from iDNA had chronic hepatitis B. It is unknown whether age, immune function, sex, or the HBsAg expressed from iDNA played a role in this difference.

Two HBeAg− chimpanzees (88A010 and 95A010) maintained serum HBV DNA near the LLOQ at baseline and again at LFU. Chimpanzees 88A010 and 95A010 had 71 and 5 cccDNA-derived transcripts, respectively, after the ETV lead-in, and had 0.002 and 0.1 copies/cell cccDNA, respectively, after the end of all treatment. Prior to siRNA treatment, chimpanzee 88A010 with the lower amount of cccDNA had more iDNA transcripts (267 total) than chimpanzee 95A010 (74 total). In both animals, these transcripts primarily encoded HBsAg. Their transcripts were not characterized at the end of treatment. Chimpanzee 95A010’s ALT was in the normal range pre-and post-study, identifying this chimpanzee as being in the immune control phase of CHB prior to and after siRNA treatment. Chimpanzee 88A010’s ALT was 1.4 × ULN pre-study and normalized post-siRNA treatment, suggesting a potential but uncharacterized benefit of siRNA treatment.

### 3.4. cccDNA Versus HBV Transcripts in siRNA-Treated HBeAg-Negative Patients

In contrast to the low amount of cccDNA in the chimpanzees that were HBeAg− at the start of the study, the HBeAg− patients (patients 701, 705, 709, and 712) that had biopsies had 0.38, 1.0, 0.26, and 1.1 copies/cell cccDNA. These amounts were equal to or 3-fold higher than the HBeAg-seroconverted chimpanzees 89A008 and A4A014. No HBV transcripts were detected in two of these patients, patient 701 whose HBsAg was 1.9 IU/mL at last follow-up and patient 709 who seroconverted for HBsAg. Only 8 and 0 cccDNA-derived transcripts were detected in patients 705 and 712, respectively. Altogether, these values suggest the transcriptional silencing of cccDNA in the HBeAg− patients treated with HBV siRNA and ETV. With only a single biopsy, however, there are no data to demonstrate whether the amount of cccDNA changed during treatment.

### 3.5. HBx Transcripts Produced from Integrated HBV in Patients

Integrated HBV fragments from the double-stranded linear DNA (dslDNA) replication by-product can produce either HBsAg or HBx. At the time of post-siRNA treatment biopsy, most of the HBV transcripts in the previously highly viremic male patient 710 (a total of 61 HBV transcripts) were from iDNA. This result is consistent with continued expression from iDNA after the transcriptional silencing of cccDNA that was observed in transitional chimpanzee 89A008. These iDNA transcripts in patients encoded truncated HBx (39%) and HBsAg (33%). The types of transcripts were similar in patient 710 who had become HBeAg− during treatment and in two patients who were HBeAg− at the start of the study (patients 705 and 712), but the proportions shifted in favor of truncated HBx transcripts (approximately 60%) and fewer HBsAg transcripts (approximately 20%) in these older patients. Many of the X transcripts were truncated at the alternative polyA, as previously described by Hilger et al. [11], and are expected to produce truncated HBx protein that retains the ability to bind DDB1 and destabilize the Smc5/6 complex [26]. The accumulation of truncated HBx promotes cell survival and clonal expansion [27], which may explain why the older patients 705 and 712 have more X transcripts than S transcripts. The preferential survival of cells expressing HBx could select for the greater expression of HBx than HBsAg. Interestingly, HBx transcripts expressed from iDNA did not prevent the silencing of the cccDNA in the ARC-520-treated patients, or such transcripts were expressed in different cells than the ones that contained cccDNA.

### 3.6. HBx Transcripts in High-Viremia HBeAg-Positive Chimpanzees

The constant expression of HBx was shown to be required for HBV antigen expression and replication [28], and the HBx mRNA has long been defined as the 0.7 kilobase canonical transcript [29]. However, we only observed a single canonical X transcript in thirteen CHB chimpanzee liver biopsy specimens. Rather than canonical X, the highly viremic HBeAg+ chimpanzees had long X transcripts as described by Stadelmayer et al. [30] with variable transcription start sites beginning after the start of the small S HBsAg ORF and ending with the HBV polyA site. The 5’ end of transcripts with the potential to encode HBx in chimpanzees had a broader range of start sites than any of the other HBV transcripts.

### 3.7. HBV Transcripts in Chimpanzees That Appear to Result from dslDNA Dimers

A total of 35 transcripts from HBeAg+ chimpanzees had two copies of X and some of these started at the canonical X start site. Guo et al. reported that the X mRNA that terminated at the HBV polyA produced the canonical 0.7 kb transcript and that a 3.9 kb transcript that resulted from leaky termination produced a transcript with two copies of X [31]. These studies were performed in human hepatocyte cell lines. Only 14% of the overlength X transcripts that we identified could have been the result of leaky termination because they had the same sequence in both copies of X. In all the rest, the two copies of X differed in sequence.

Rather than being the product of leaky termination, we proposed that these overlength X transcripts were the result of the transcription of dslDNA dimers that had ligated to each other and circularized. HBV quasi-species are known to often have insertions or deletions in the X ORF, which is between DR2 and DR1 [32]. The most direct source of such species is likely to be dslDNA with DR1 at each end that self-ligated, such as those that form quasi-species with small gaps between DR2 and DR1. It stands to reason that dimers of dslDNA with DR1 at each end could alternatively be combined by homologous recombination and then the dimers self-ligate. During active replication, the number of dslDNA molecules in the nucleus is likely to be relatively high, providing substrate for such a reaction.

HBeAg− chimpanzees also produced HBV transcripts that appeared to be the result of the ligated dimers of dslDNA [12]. These transcripts, however, encoded HBsAg rather than HBx and had larger sequence gaps between the putative dimers. In this case, much of the X ORF was deleted. The HBeAg− chimpanzees had far less replication than the HBeAg+ chimpanzees, which would have resulted in fewer numbers of dslDNA molecules, and these could more readily have the nuclease digestion of the ends prior to encountering another molecule with which to ligate.

While the presence of overlength HBV transcripts from putative dslDNA dimers in chimpanzee livers was intriguing, such transcripts were not observed in any of our patient samples. Circularized dimers would be a form of cccDNA and the transcription of these would, thus, also be inhibited by the Smc5/6 complex. The full-length sequencing of liver HBV transcripts of actively transcribing HBeAg+ CHB patients would be the necessary source to determine whether such transcripts exist in patients.

## 4. Conclusions and Discussion

ARC-520 was a first-in-class siRNA therapeutic for chronic HBV infection that brought to light striking differences in the HBV virology between the HBeAg+ and HBeAg− chimpanzees, and these differences were reflected in the patients even after a single dose of ARC-520 [7]. The key differences were due to HBsAg primarily being produced from iDNA in HBeAg− individuals. The most important finding from the long-term ARC-520 study in the patients was that the products of cccDNA expression became dramatically reduced or lost following siRNA treatment in conjunction with ETV [12,18].

The finite HBV siRNA treatment of CHB patients concomitant with NA replication inhibition has demonstrated a durable reduction in HBsAg, HBeAg, HBcrAg, and HBV RNA in some patients following siRNA treatment for as short as three months or up to 11 months [18,20,33,34]. These results were observed in patients treated with ARC-520 and in patients treated with the follow-on GalNAc-targeted HBV siRNA therapeutic daplusiran/tomligisiran (previously JNJ-3989) that did not include the excipient EX1. Such reductions were durable in some patients [18,20].

HBV transcriptional analysis and the cccDNA quantification of the Heparc-2001 patients demonstrated that the mechanism whereby the patients treated with ARC-520 siRNA were able to achieve long-term reduction in viral parameters was the transcriptional silencing of the cccDNA. The HBx protein promotes the transcription of cccDNA by causing the degradation of the structural maintenance of chromosome 5/6 complex (SMC5/6) [28]. Allweiss et al. observed that siRNA targeting HBx was able to restore the SMC5/6 complex and suppress the transcription of cccDNA [35]. Their study was conducted in human liver chimeric mice. The maintenance of the silencing to prevent the post-siRNA rebound in these mice required the prevention of new HBV infection, which the authors blocked by treating the mice with the entry inhibitor myrcludex-B. Balogopal et al. have observed transcriptionally silent cccDNA in HBV/HIV-co-infected patients treated with NA [36]. The combination of ARC-520 siRNA and NA yielded transcriptional silencing in the HBeAg+ patients that serve as case studies, given that Heparc-2001 contained only three HBeAg+ patients for whom ARC-520 was suitable to yield deep reductions in viral transcripts and their products.

The paucity of cccDNA-derived products in the HBeAg− patients suggests that their cccDNA was also silenced, and we were unable to detect any HBV transcripts by Iso-seq from the HBeAg− patient who lost HBsAg. The amounts of cccDNA in these HBeAg− patients were equivalent to the quantity in one chimpanzee who had chronic hepatitis with high viremia (chimpanzee 89A008). However, these HBeAg− patients remain on ETV treatment and we do not know whether the cccDNA would remain durably silenced without ETV.

All three patients in whom we could detect full-length HBV transcripts by Iso-seq had truncated HBx transcripts. Truncated HBx protein, as from transcripts terminated at the alternative polyA, is known to retain the ability to bind DDB1 and suppress SMC5/6 activity [37,38]. Aggarwal et al. noted that CHB patients had a pattern of diffuse hepatocytes positive for core antigen, presumably expressing cccDNA, and the foci of HBsAg-positive cells expected to contain integrated HBV [39]. They rarely observed cells expressing both core and HBsAg. Given that the Heparc-2001 patients expressed both HBsAg and HBx from iDNA, the more probable explanation for truncated HBx not preventing the transcriptional silencing of cccDNA is that the iDNA and cccDNA were in different cells.

siRNA plus NA alone may not be adequate to bring about the silencing; these patients received repeat siRNA dosing separated by intervals of NA alone. Two HBeAg+ and one HBeAg− patient responded similarly to the intervals of RNAi + ETV and ETV alone. Patients’ age was observed to be negatively correlated with a long-term reduction in viral parameters following siRNA treatment [20]. HBeAg+ patients tend to be younger, and younger patients have a less exhausted T-cell response [40]. Interestingly, Ganchua et al. observed increased cytokine signatures, increased T cell activation, and a less exhausted phenotype in patients following HBsAg reduction with the siRNA AB-729 [41,42].

The limitations of the ARC-520 studies were that the number of patients was small, and the chimpanzee study design differed from that of the patient study. Chimpanzees could not be maintained on ETV and follow-up analyses in the chimpanzees were prohibited by their reclassification as endangered. The patients received a single biopsy at LFU but there were no pre-study biopsies for comparison.

Although ARC-520 development has been discontinued, multiple HBV siRNA therapeutics are now in clinical development [43]. The promising observation that the transcriptional silencing of cccDNA can be achieved will need to be repeated in larger studies to determine how best to achieve this result in more patients.

## Figures and Tables

**Table 1:** Combined patient study results. The baseline for the patients in study Heparc-2001 was prior to receiving their first dose of entecavir (ETV). This was the same day they received the single dose (SD) of ARC-520. Serum HBV parameters were available for all eight patients, but biopsy data were not available (N/A) for three patients. The last follow-up (LFU) for patients was ~30 months after the last multi-dose (MD) ARC-520. cccDNA measurement from patient liver biopsies was at their LFU. HBV transcripts were identified by single-molecule real-time (SMRT) sequencing (Iso-seq) and characterized as described [12]. Transcript counts that terminated at the well-defined HBV polyadenylation signal (polyA) were identified as cccDNA-derived, and those that terminated prior to this polyA, including at the alternative polyA signal, were identified as being derived from integrated HBV DNA (iDNA). The iDNA-derived transcripts usually included some host sequence at the 3-prime terminus, even when they terminated following the alternative polyA.

Patient/ HBeAg	HBsAg (IU/mL)		HBV DNA (log_10_ IU/mL)		HBeAg (PEI U/mL)	cccDNA (Copies/Cell)	HBV Transcripts at LFU	
	Baseline	LFU	Baseline	LFU	LFU	LFU	cccDNA-Derived	iDNA-Derived
708/Pos	34,388	<LLOQ	8.54	TND	<LLOQ	N/A	N/A	N/A
710/Pos	80,918	25.8	8.85	<LLOQ	<LLOQ	3.425	7	44
711/Pos	64,167	1113	8.69	1.79	6.57	N/A	N/A	N/A
701/Neg	6.87	1.92	3.41	TND	<LLOQ	0.382	TND	TND
705/Neg	4085	2677	5.16	TND	<LLOQ	1.025	8	104
706/Neg	1256	425	4.26	<LLOQ	<LLOQ	N/A	N/A	N/A
709/Neg	10	<LLOQ	3.76	<LLOQ	<LLOQ	0.263	TND	TND
712/Neg	2016	1018	4.37	TND	<LLOQ	1.085	0	57

LFU, last follow-up; Pos, HBeAg+ at start of study; Neg, HBeAg− at start of study; LLOQ, lower level of quantitation TND, target not detected; N/A, not available.

**Table 2:** Combined chimpanzee study results. Baseline (study day 1) for the chimpanzees was prior to receiving their first dose of ETV. LFU for serum HBV parameters in the chimpanzees was study day 533, seven months after the last dose of ETV. cccDNA measurements in chimpanzees are shown at the end of the 8–20 weeks ETV lead-in period (“ETV Lead-in”), at the nadir of the response to siRNA + ETV treatment (“SiRNA + ETV”), and 28–61 days off all treatment when replication had rebounded (“Post-siRNA, No ETV”). HBV transcript counts for HBeAg+ chimpanzees are shown for the pre-study health check (HC) that was 40 days prior to baseline to represent their naïve state. HBV transcript counts for the HBeAg-negative chimpanzees were measured only once, after the 57-day ETV lead-in; prior RNA-seq analysis had demonstrated virtually identical transcript histograms at HC and after the ETV lead-in for these HBeAg− chimpanzees [7]. Transcripts at HC and at study day 1 remained similar for all chimpanzees except chimpanzee 89A008 who transitioned to HBeAg− during the ETV lead-in (see Supplementary Figure S5 in Wooddell et al. [7]).

Chimpanzee/ HBeAg	HBsAg (µg/mL)		HBV DNA (log_10_ Copies/mL)		HBeAg (ng/mL)	cccDNA (Copies/Cell)			HBV Transcripts Prior to SiRNA	
	Baseline	LFU	Baseline	LFU	LFU	ETV Lead-in	SiRNA + ETV	Post-siRNA No ETV	cccDNA-Derived	iDNA-Derived
A2A004/Pos	3188	6770	8.6	8.2	17,858	1.92	0.39	9.06	2206	31
A3A006/Pos	2104	2053	8.3	8.3	8518	1.22	0.71	9.57	1831	32
A4A014/Pos	253	4.8	7.7	<LLOQ	<LLOQ	0.226	0.011	0.032	449	32
89A009/Pos	245	183	8.6	8.4	<LLOQ	0.072	0.012	0.033	373	208
88A010/Neg	199	251	<LLOQ	3.1	<LLOQ	0.0009	0.0007	0.0023	71	267
95A010/Neg	86	74	3.5	4.0	<LLOQ	0.0051	0.0013	0.0124	5	74

LFU, last follow-up; Pos, HBeAg+ at start of study; Neg, HBeAg− at start of study; LLOQ, lower level of quantitation.

## Data Availability

The data presented in these studies are compiled from openly available sources within prior publications and their supplementary materials to bring together the information from long-term studies in patients and chimpanzees that were published in multiple articles: https://doi.org/10.1126/scitranslmed.aan0241; https://doi.org/10.1136/gutjnl-2020-323445; https://doi.org/10.3390/v13040581; and https://doi.org/10.3390/v16121943. This is a summary of previously published data.
